# Commentary on: The actin bundling activity of ITPKA mainly accounts for its migration-promoting effect in lung cancer cells

**DOI:** 10.1042/BSR20230057

**Published:** 2023-09-11

**Authors:** Xin Zhang, Jiadi He, Dong Ren

**Affiliations:** 1 Postdoctoral Innovation Practice Base, Postdoctoral Research Center of Jiangmen Central Hospital, Southern Medical University, Jiangmen 529030, China; 2 Clinical Experimental Center, Jiangmen Key Laboratory of Clinical Biobank and Translational Research, Jiangmen Central Hospital, Jiangmen 529030, China; 3 Department of Pathology, University of California Irvine Medical Center, Orange, CA 92868, U.S.A.

**Keywords:** 1,4, 5-triphosphate 3-kinase A, 5-trisphosphate, actin, inositol 1,4, lung cancer, metastasis

## Abstract

1,4,5-triphosphate 3-kinase A (ITPKA) was first described and characterized by Irvine *et al*. in 1986 and cloned by Takazawa et al. in 1990. It is one of the components of the Ca^2+^ and calmodulin signaling pathway and a substrate for cAMP-dependent kinase (PKA) and protein kinase C (PKC), and is mainly involved in the regulation of intracellular inositol polyphosphate signaling molecules. Through a series of studies, Sabine’s team has found that ITPKA expression was up-regulated in a variety of cancer cells, and silencing ITPKA inhibited while overexpressing ITPKA promoted cancer cell migration *in vitro* and metastasis *in vivo*. The latest research from Sabine’s team has demonstrated that in H1299 lung cancer cells, the mechanism by which ITPKA promoted migration and invasion was predominantly depending on the ability of binding to F-actin, which will induce cancer cells to form a tight flexible actin networks. Small molecule compounds targeting the IP_3_ kinase activity of ITPKA protein may only inhibit the migration and invasion of cancer cells caused by the enhanced ITPKA kinase activity under ATP stimulation, but not the cytoskeletal remodeling caused by the binding of ITPKA protein to F-actin and the driven migration and invasion of cancer cells. Therefore, targeted therapeutic strategy focusing on blocking the binding of ITPKA to F-actin is indispensable when designing the inhibitors targeting ITPKA protein.

Lung cancer is the leading cause of cancer-related deaths worldwide [1]. The pathological classification of lung cancer primarily comprises two types: non-small cell lung cancer, which includes lung adenocarcinoma and lung squamous cell carcinoma and accounts for 75% of cases, and small cell lung cancer, which constitutes the mainly remaining cases [2]. In the recent years, with the development of high-throughput sequencing and targeted drugs, the prognosis of lung cancer patients has significantly improved [1]. However, pathological subtypes of lung cancer, such as squamous cell carcinoma and small cell lung cancer, remain lack of mutation sites and corresponding target, which significantly affects the outcome and survival of patients [3]. When tumor cells are not responsive to chemotherapeutic drugs, they are inclined to metastasize throughout the body, ultimately resulting in death due to multiple organ failure [4]. This finding prompts us to further investigate additional targets for drug action and enhance the scope of clinical therapeutic alternatives.

The prime targets of commonly used drugs in the clinical treatment of lung cancer function the anti-tumor role by blocking growth signals, inhibiting core signaling pathways, damaging DNA, inhibiting key metabolic enzymes, blocking the cell cycle, remodeling the microenvironment, destroying abnormal angiogenesis, and relieving immunosuppression [5–7]. However, the drugs targeting the movement of cancer cells seems to be scant. The underlying reasons include the absence of specific mechanisms explaining the movement of cancer cells, extensive crosstalk with the molecular network contributing to cell movement, and the lack of a clear pharmacodynamic hub for the skeleton proteins involved in cell movement. Therefore, in-depth understanding and investigation of the mechanism of cancer cell movement regulation is of great necessity for the identification of potential therapeutic targets.

Cytoskeletal proteins are mainly divided into three categories: microtubules, microfilaments and intermediate fibers, and the basis of cell motility is the polymerization and depolymerization of cytoskeletal proteins [8]. Actin is the structural protein of microfilaments, and the monomer is called globular actin (G-actin) and the polymer is called filamentous actin (F-actin) [9]. G-actin can polymerize into F-actin under ATP supply and Mg^2+^ catalysis, while F-actin can spontaneously depolymerize under Ca^2+^ catalysis [9]. Therefore, G-actin and F-actin are in a dynamic process of polymerization and depolymerization in normal biological process, which is pivotal for the formation or degradation of microfilaments in the specific region [8,9]. The actin cytoskeleton is extensively involved in a multitude of cellular processes, including the positioning of intracellular organelles, cytoplasmic division, establishment of cell polarity, formation of pseudopods and adherent patches [10]. Moreover, it is closely associated with the division and motility of cancer cells and presents a potential therapeutic target.

The dynamic process of microfilament polymerization and depolymerization is influenced by calcium signals, so inositol 1,4,5-trisphosphate (IP_3_), a second messenger molecule capable of mobilizing Ca^2+^ release from the endoplasmic reticulum, is one of the key regulatory molecules [11]. The extracellular signaling molecule binds to the corresponding G protein-coupled receptor and transmits the activation signal to phospholipase Cβ (PLCβ) via the activated q type of Gα subunit (Gαq) [12]. PLCβ divides phosphatidyl-inositol-4,5-bisphosphate (PIP_2_) into two intracellular second messengers: diacylglycerol (DAG) and IP_3_ are subsequently activating protein kinase C (PKC) and IP_3_ receptors, respectively [13]. The IP_3_ receptor is located on the endoplasmic reticulum (ER), and activates its coupled ligand-gated calcium channels by binding to IP_3_, which further triggers the release of large amounts of Ca^2+^ into the cytoplasm and gives rise to the inward flow of extracellular Ca^2+^ [14,15].

The 1,4,5-triphosphate 3-kinase A (ITPKA) is one of the components of the Ca^2+^ and calmodulin signaling pathway and a substrate for cAMP-dependent kinase (PKA) and PKC, and is mainly involved in the regulation of intracellular inositol polyphosphate signaling molecules [16,17]. ITPKA can phosphorylate IP3 to inositol 1,3,4,5-trisphosphate (IP_4_), which can be further phosphorylated to inositol 1,3,4,5,6-pentakisphosphate (IP_5_) by inositol polyphosphate multikinase (IPMK) or dephosphorylated to inositol 1,4-bisphosphate (IP_2_) by inositol polyphosphate-4-phosphatase (INPP4) [16,18,19]. Since the affinity of INPP4 for IP_4_ is approximately 10-fold higher than that of IP_3_, the increased concentration of IP_4_ will protect IP_3_ from dephosphorylation, where the duration and concentration of IP_3_-mediated Ca^2+^ signaling depends on the ITPKA/INPP4 activity ratio [16,19,20].

Through a series of studies [16,21–23], Sabine’s team found that ITPKA expression was up-regulated in a variety of cancer cells, and silencing ITPKA inhibited cancer cell migration *in vitro* and metastasis *in vivo*, while overexpressing ITPKA promoted these processes. By comparing cancer tissues from different sources, Sabine’s team found that ITPKA expression was higher in lung adenocarcinoma, correlated with poor clinical and pathological staging in patients, and predicted poor survival outcomes in these patients [23], all of which could be verified in online open databases (Figure 1). Relevant mechanistic studies have shown that ITPKA promoted cancer cell migration through two different mechanisms: (1) without growth factor stimulation, highly expressed ITPKA binds to F-actin through the N-terminal actin-binding domain (ABD), increasing the polymerization level of cellular F-actin to stabilize microfilaments and subsequently promoting formation of large cellular protrusions [21]. (2) Under growth factor stimulation, ITPKA in cells can increase IP_4_ content through enzymatic activity, protect IP_3_ from dephosphorylation, enhance the duration and concentration of Ca^2+^ signaling mediated by IP_3_, and thus enhance ITPKA-induced migration [21]. Also, Sabine’s team has found that impaired repressor-element-1-silencing transcription factor (REST)/neuron-restrictive silencer factor (NRSF) repression of gene expression function in cancer cells relieved the state of ITPKA expression repression, leading to its aberrant high expression in cancer cells [22]. In addition, Sabine’s team examined ITPKA expression in a variety of solid tumor tissues and cells and found that ITPKA expression was most significantly up-regulated in lung cancer and further highly expressed in metastatic lymph node, suggesting that ITPKA may be a potential target for anti-metastasis therapy against lung cancer [23].

**Figure 1:**
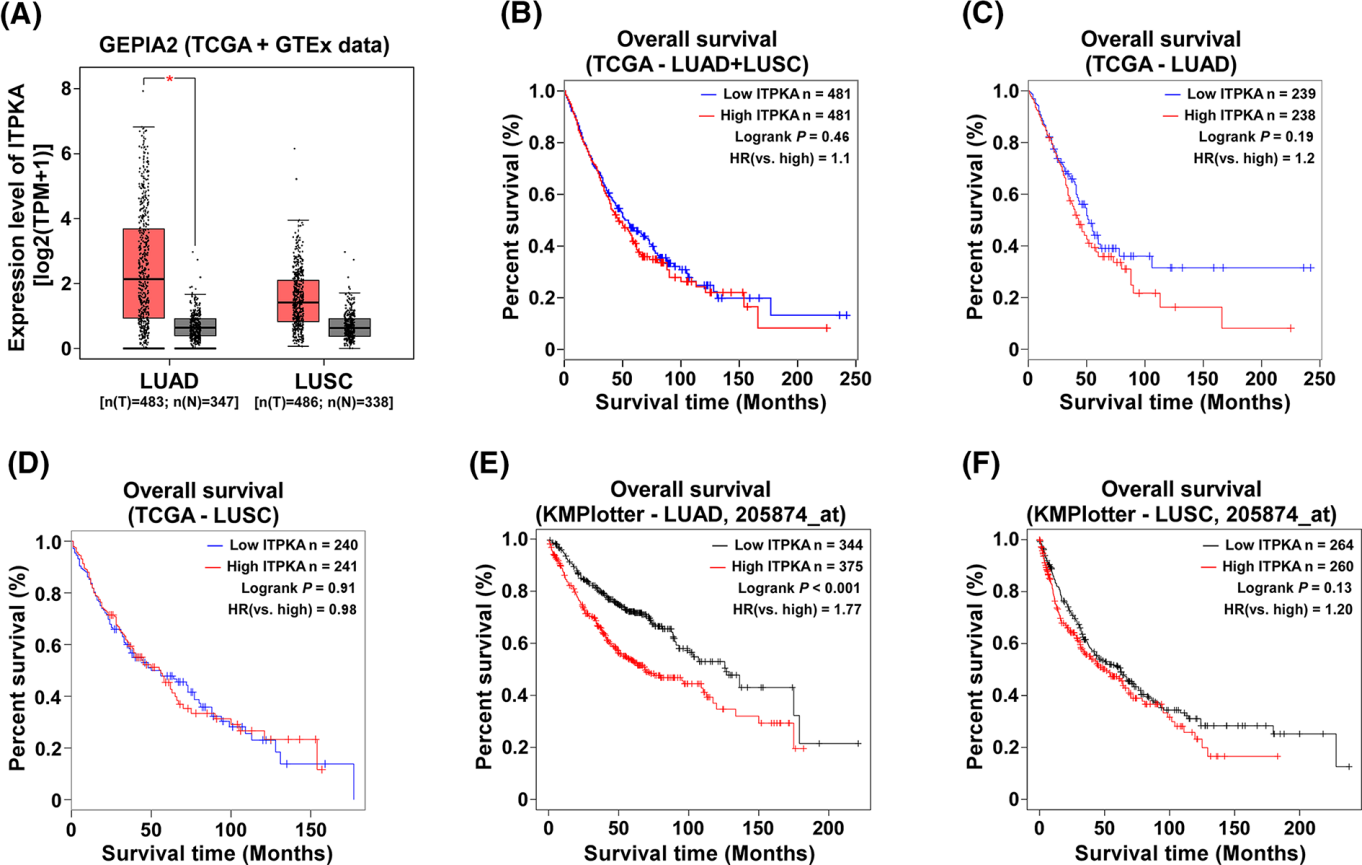
Online open databases have shown that ITPKA is highly expressed in lung adenocarcinoma tissues and predicts a worse prognosis for patients (**A**) Compared with normal lung tissue (N), ITPKA expression was increased in lung adenocarcinoma (LUAD), but not significantly up-regulated in lung squamous cell carcinoma (LUSC). (**B–D**) In the TCGA database, high expression of ITPKA in LUAD predicted poor survival prognosis of patients (**C**), while there was no trend in overall lung cancer (B) or LUSC (D). (**E,F**) In the KMplotter database, high ITPKA expression in LUAD predicted poor prognosis in patients (E), while there was no correlation in LUSC (F). The data in (A–D) come from GEPIA2 (http://gepia2.cancer-pku.cn/) and the data in (E,F) come from Kaplan–Meier Plotter (http://kmplot.com/).

The mutation rate of ITPKA is low, approximately 1.1%, in pan-cancer tissue (Figure 2A). Mutation types include missense and truncating variants, with the majority localized to the IPK region (Figure 2B). The ITPKA gene, comprising seven exons, is situated on the 15th chromosome, and it spans a length of 1825 nucleotides, with a coding sequence that initiates at position 55 and concludes at position 1440 (Figure 2B). There are 461 amino acid (aa) in ITPKA in ITPKA and aa 245-455 belongs to the IPA region, and Lys264 plays a critical role in the catalytic mechanism of IP3-3K (Figure 2B,C). This residue interacts with the ligands in the substrate complex and the 3-phosphate in the product complex, potentially aiding the nucleophilic attack of the phosphate by neutralizing the transition state’s negative charge and directing the phosphate group transfer (Figure 2D). This function is analogous to Lys168 in PKA [24]. PKA also possesses another crucial catalytic residue, Asp166, which forms a tighter interaction with the serine hydroxyl. Asp166 primarily functions to choose the appropriate stereochemical isomer of the serine hydroxyl group in the substrate, thus enabling and enhancing the nucleophilic attack by the hydroxyl group [24]. Previous research teams claim that the N-terminal domain aa 1–52 of ITPKA binds actin filaments [25]. AlphaFold2 was used to predicted the 3D structure of the full length of ITPKA. Residues 1–197 constitute a flexible disordered domain with no fixed structure, characterized by lower pLDDT scores (Figure 2E). However, residues 31–50 are capable of forming an alpha helix. To confirm the existence of this structure and its involvement in actin binding, further experimental validation by the research team is required.

**Figure 2:**
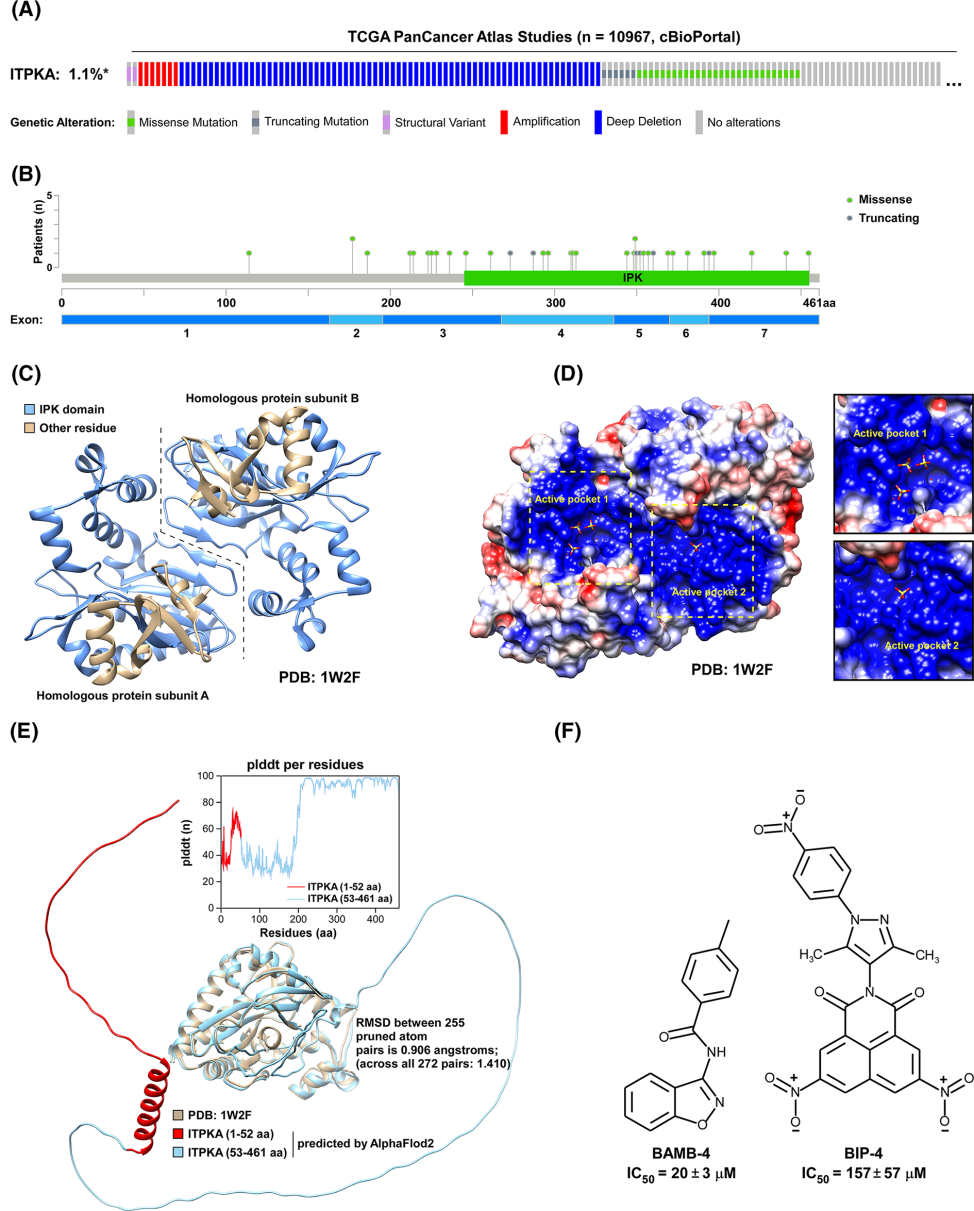
The gene and protein structures of ITPKA (**A**) Based on 10,967 Pancancer Atlas studies in the TCGA database within cBioPortal, the altered/profiled rate of ITPKA is 115/10,950, which corresponds to 1.1%. (**B**) The ITPKA gene consists of seven exons, with missense and truncating mutations being the most prevalent types. These mutations primarily occur in the IPK region. (**C**) ITPKA functions as a homodimer, where the IPK domain is represented in blue, and the remaining portions are shown in yellow. The PDB (1W2F) structure spans amino acids 185–461, and homologous subunits are indicated by dashed lines. (**D**) The colored surface represents the electrostatic potential, where red indicates a negative charge and blue indicates a positive charge. The two active pocket domains, which are positively charged, interact with ligands. (**E**) The structure of ITPKA predicted by AlphaFold2 (AF) (cyan) aligns well with the structure from the Protein Data Bank (yellow). The AF structure reveals that amino acids 1–185 form a flexible loop with a low plddt value. Additionally, the Schröder team found that amino acids 1–52 (red) bind to the actin filament, and there is a suggestion that amino acids 31–50 form an alpha helix. (**F**) The Sabine team discovered two compounds that act as inhibitors for ITPKA. Among these, BIP-4 demonstrated a better inhibitory effect with an IC_50_ value of 157 ± 57 μM compared with BAMB-4.

The Sabine research team conducted a high-throughput screen of 341,440 small molecule compounds using an ADP-Glo assay to identify potential inhibitors of ITPKA [26]. The initial screen yielded 237 hit compounds exhibiting inhibitory activity. The inhibitory effects of MEPTT-3 and BAMB-4 on ITPKA activity were validated using an orthogonal assay (coupled PK/LDH optical assay). The results showed that MEPTT-3 and BAMB-4 had IC50 values of 35 ± 4 μM and 20 ± 3 μM, respectively. Cellular uptake experiments showed that BAMB-4 could be completely absorbed by lung cancer H1299 cells and remained stable after cellular uptake. The above evidence indicates that BAMB-4 is a druggable inhibitor of ITPKA [26]. To gain a deeper understanding of BAMB-4, its mechanism of inhibition and kinetic parameters were investigated. It was revealed that BAMB-4 acts as a mixed-type inhibitor, affecting both ATP and IP3 binding. During their investigation, BAMB-4 demonstrated an IC50 value of 20 μM for inhibiting InsP3 kinase activity. In another research from the Sabine team [27], they found a novel InsP3 kinase inhibitor, named BIP-4, with an IC50 of 157 ± 57 nM, exhibiting stronger inhibitory activity compared with the previous inhibitor BAMB-4. In contrast, BIP-4 competitively obstructs InsP3 binding to ITPKA but does not interfere with ATP binding. It occupies the binding site, overlapping with InsP3, resulting in selective inhibition. BIP-4 enters cells through non-endocytic mechanisms and is capable of inhibiting ITPKA within the cellular environment. However, clinical trials of the aforementioned compounds have not been observed, and their pharmaceutical properties still need further evaluation.

When studying the specific molecular mechanism of ITPKA and F-actin interaction, Sabine’s team further found that ITPKA not only binds to F-actin through the N-terminal ABD but also that the C-terminal IP3 kinase domain is involved in the regulation of F-actin arrangement. This enables ITPKA to form tight and flexible actin networks and facilitates the formation of cellular pseudopods [28]. This finding supported the notion that the mechanism underlying the ITPKA regulation of cancer cell migration is a multi-pathway and multi-levels process, and the effect of related mechanisms on cancer cell metastasis needs to be literally investigated. To elucidate those mechanisms involved, Sabine’s team performed the following experimental treatments in their latest research paper published in *Bioscience Reports* [19]: (1) inhibited the actin-binding regulatory activity of endogenous ITPKA using an ITPKA mutant overexpressing an interaction structural domain deletion (ITPKA^L34P^); (2) inhibited the actin-binding regulatory activity of endogenous ITPKA using an ITPKA mutant overexpressing an IP_3_ catalytic activity loss ITPKA mutant (ITPKA^D416N^) or addition of IP_3_ kinase inhibitor GNF362, to observe the alteration of IP_3_ catalytic activity of ITPKA on the migration and invasion ability of lung cancer cells under normal or ATP-stimulated conditions.

In the present study [19], Sabine's team observed two important results: (1) Overexpression of ITPKA did not increase the migration and invasion of lung cancer H1299 cells under normal culture conditions; however, overexpression of ITPKA^L34P^ significantly inhibited the migration of cancer cells and decreased the motility of filamentous pseudopods. (2) Overexpression of ITPKA slightly increased the migratory ability of cancer cells under ATP stimulation, but overexpression of ITPKA^D416N^ had no significant effect, while GNF362 inhibited the migratory ability of cancer cells increased by ITPKA.

In conclusion, the data from Sabine’s team suggested that in H1299 lung cancer cells, the mechanism by which ITPKA promoted migration and invasion was predominantly depend on the ability of binding to F-actin, which induces cancer cells to form a tight flexible actin networks [19]. Small molecule compounds targeting the IP_3_ kinase activity of ITPKA protein may only inhibit the migration and invasion of cancer cells caused by the enhanced ITPKA kinase activity under ATP stimulation, but not the cytoskeletal remodeling caused by the binding of ITPKA protein to F-actin and the driven migration and invasion of cancer cells [19]. Therefore, the most important implication of the present study for subsequent research is that targeted therapeutic strategy focusing on blocking the binding of ITPKA to F-actin is indispensable when designing the inhibitors targeting ITPKA protein.

Overall, the findings in the present study from Sabine’s team exhibit excellent sustainability. In the meantime, the present study offers novel scientific ideas for the development of ITPKA inhibitors.

## Data Availability

Online open databases were not generated nor owned by the authors and are available online. RNA expression levels of ITPKA in lung adenocarcinoma (LUAD) and squamous cell carcinoma (LUSC) and their correlation with survival and prognosis were obtained from database GEPIA2 (http://gepia2.cancer-pku.cn/) and Kaplan–Meier Plotter (http://kmplot.com/), respectively. Mutation information is sourced from the TCGA PanCancer Atlas Studies within cBioPortal (https://www.cbioportal.org/). The 3D crystal structure, identified by PDB ID 1w2f, originates from the Protein Data Bank. AlphaFold2 (Colab version) was utilized to predict ITPKA’s complete 3D structure [29,30]. The electrostatic surface was calculated using DelPhi and visualized in UCSF Chimera [31,32]. All relevant data are provided within the manuscript, figures and supplementary data. Other data used and/or analyzed during the present study are available from the corresponding author on reasonable request.

## Abbreviations

ABD: actin-binding domain
AF: AlphaFold2
DAG: diacylglycerol
ER: endoplasmic reticulum
F-actin: filamentous actin
G-actin: globular actin
Gαq: q type of G protein α subunit
INPP4: inositol polyphosphate-4-phosphatase
IP_2_: inositol 1,4-bisphosphate
IP_3_: inositol 1,4,5-trisphosphate
IP_4_: inositol 1,3,4,5-trisphosphate
IP_5_: 1,3,4,5,6-pentakisphosphate
IPMK: inositol polyphosphate multikinase
NRSF: neuron-restrictive silencer factor
PDB: Protein Data Bank
PIP_2_: phosphatidyl-inositol-4,5-bisphosphate
PKA: cAMP-dependent kinase
PKC: protein kinase C
PLCβ: phospholipase Cβ
REST: repressor-element-1-silencing transcription factor
TCGA: The Cancer Genome Atlas

## References

[1] Siegel R.L., Miller K.D., Wagle N.S. and Jemal A. (2023) Cancer statistics, 2023. CA Cancer J. Clin. 73, 17–48 10.3322/caac.21763 36633525

[2] Li Y., Zhang X., Cai J., Ren L., Liu B., Wu M. et al. (2022) The pathological tissue expression pattern and clinical significance of m6A-regulatory genes in non-small cell lung cancer. J. Gene Med. 24, e3397 10.1002/jgm.3397 34751492

[3] Chen J., Liu A., Wang Z., Wang B., Chai X., Lu W. et al. (2020) LINC00173.v1 promotes angiogenesis and progression of lung squamous cell carcinoma by sponging miR-511-5p to regulate VEGFA expression. Mol. Cancer 19, 98 10.1186/s12943-020-01217-2 32473645 PMC7260858

[4] Nussinov R., Tsai C.J. and Jang H. (2021) Anticancer drug resistance: an update and perspective. Drug Resist. Updat. 59, 100796 10.1016/j.drup.2021.100796 34953682 PMC8810687

[5] Zhang Z., Bu L., Luo J. and Guo J. (2022) Targeting protein kinases benefits cancer immunotherapy. Biochim. Biophys. Acta Rev. Cancer 1877, 188738 10.1016/j.bbcan.2022.188738 35660645

[6] Gourley C., Balmana J., Ledermann J.A., Serra V., Dent R., Loibl S. et al. (2019) Moving from Poly (ADP-Ribose) polymerase inhibition to targeting DNA repair and DNA damage response in cancer therapy. J. Clin. Oncol. 37, 2257–2269 10.1200/JCO.18.02050 31050911

[7] Chen S., Zhao Y., Liu S., Zhang J., Assaraf Y.G., Cui W. et al. (2022) Epigenetic enzyme mutations as mediators of anti-cancer drug resistance. Drug Resist. Updat. 61, 100821 10.1016/j.drup.2022.100821 35219075

[8] Mohapatra S. and Wegmann S. (2023) Biomolecular condensation involving the cytoskeleton. Brain Res. Bull. 194, 105–117 10.1016/j.brainresbull.2023.01.009 36690162

[9] Rajan S., Kudryashov D.S. and Reisler E. (2023) Actin bundles dynamics and architecture. Biomolecules 13, 450 10.3390/biom13030450 36979385 PMC10046292

[10] Povarova O.I., Antifeeva I.A., Fonin A.V., Turoverov K.K. and Kuznetsova I.M. (2023) The role of liquid-liquid phase separation in actin polymerization. Int. J. Mol. Sci. 24, 3281 10.3390/ijms24043281 36834689 PMC9961026

[11] Finkelstein M., Etkovitz N. and Breitbart H. (2020) Ca(2+) signaling in mammalian spermatozoa. Mol. Cell. Endocrinol. 516, 110953 10.1016/j.mce.2020.110953 32712383

[12] Maziarz M., Federico A., Zhao J., Dujmusic L., Zhao Z., Monti S. et al. (2020) Naturally occurring hotspot cancer mutations in Galpha(13) promote oncogenic signaling. J. Biol. Chem. 295, 16897–16904 10.1074/jbc.AC120.014698 33109615 PMC7864081

[13] Katan M. and Cockcroft S. (2020) Phosphatidylinositol(4,5)bisphosphate: diverse functions at the plasma membrane. Essays Biochem. 64, 513–531 10.1042/EBC20200041 32844214 PMC7517351

[14] Ermakov A., Daks A., Fedorova O., Shuvalov O. and Barlev N.A. (2018) Ca(2+) -depended signaling pathways regulate self-renewal and pluripotency of stem cells. Cell Biol. Int. 42, 1086–1096 10.1002/cbin.10998 29851182

[15] Zhang X., Zhang L., Lin B., Chai X., Li R., Liao Y. et al. (2017) Phospholipid phosphatase 4 promotes proliferation and tumorigenesis, and activates Ca(2+)-permeable cationic channel in lung carcinoma cells. Mol. Cancer 16, 147 10.1186/s12943-017-0717-5 28851360 PMC5576330

[16] Windhorst S., Song K. and Gazdar A.F. (2017) Inositol-1,4,5-trisphosphate 3-kinase-A (ITPKA) is frequently over-expressed and functions as an oncogene in several tumor types. Biochem. Pharmacol. 137, 1–9 10.1016/j.bcp.2017.03.023 28377279 PMC5555585

[17] Erneux C., Roeckel N., Takazawa K., Mailleux P., Vassart G. and Mattei M.G. (1992) Localization of the genes for human inositol 1,4,5-trisphosphate 3-kinase A (ITPKA) and B (ITPKB) to chromosome regions 15q14-q21 and 1q41-q43, respectively, by in situ hybridization. Genomics 14, 546–547 10.1016/S0888-7543(05)80265-4 1330886

[18] Leyman A., Pouillon V., Bostan A., Schurmans S., Erneux C. and Pesesse X. (2007) The absence of expression of the three isoenzymes of the inositol 1,4,5-trisphosphate 3-kinase does not prevent the formation of inositol pentakisphosphate and hexakisphosphate in mouse embryonic fibroblasts. Cell. Signal. 19, 1497–1504 10.1016/j.cellsig.2007.01.024 17355905

[19] Küster L., Paraschiakos T., Karakurt K.E., Schumacher U., Diercks B.P. and Windhorst S. (2023) The actin bundling activity of ITPKA mainly accounts for its migration-promoting effect in lung cancer cells. Biosci. Rep. 43, BSR20222150 10.1042/BSR20222150 36688944 PMC9912108

[20] Windhorst S., Minge D., Bahring R., Huser S., Schob C., Blechner C. et al. (2012) Inositol-1,4,5-trisphosphate 3-kinase A regulates dendritic morphology and shapes synaptic Ca2+ transients. Cell. Signal. 24, 750–757 10.1016/j.cellsig.2011.11.010 22120525

[21] Windhorst S., Fliegert R., Blechner C., Möllmann K., Hosseini Z., Günther T. et al. (2010) Inositol 1,4,5-trisphosphate 3-kinase-A is a new cell motility-promoting protein that increases the metastatic potential of tumor cells by two functional activities *. J. Biol. Chem. 285, 5541–5554 10.1074/jbc.M109.047050 20022963 PMC2820782

[22] Chang L., Schwarzenbach H., Meyer-Staeckling S., Brandt B., Mayr G.W., Weitzel J.M. et al. (2011) Expression regulation of the metastasis-promoting protein InsP3-kinase-A in tumor cells. Mol. Cancer Res. 9, 497–506 10.1158/1541-7786.MCR-10-0556 21460179

[23] Windhorst S., Kalinina T., Schmid K., Blechner C., Kriebitzsch N., Hinsch R. et al. (2011) Functional role of inositol-1,4,5-trisphosphate-3-kinase-A for motility of malignant transformed cells. Int. J. Cancer 129, 1300–1309 10.1002/ijc.25782 21792881

[24] González B., Schell M.J., Letcher A.J., Veprintsev D.B., Irvine R.F. and Williams R.L. (2004) Structure of a human inositol 1,4,5-trisphosphate 3-kinase: substrate binding reveals why it is not a phosphoinositide 3-kinase. Mol. Cell. 15, 689–701 10.1016/j.molcel.2004.08.004 15350214

[25] Schell M.J., Erneux C. and Irvine R.F. (2001) Inositol 1,4,5-trisphosphate 3-kinase A associates with F-actin and dendritic spines via its N terminus *. J. Biol. Chem. 276, 37537–37546 10.1074/jbc.M104101200 11468283

[26] Schröder D., Rehbach C., Seyffarth C., Neuenschwander M., Kries J.V. and Windhorst S. (2013) Identification of a new membrane-permeable inhibitor against inositol-1,4,5-trisphosphate-3-kinase A. Biochem. Biophys. Res. Commun. 439, 228–234 10.1016/j.bbrc.2013.08.053 23981806

[27] Schröder D., Tödter K., Gonzalez B., Franco-Echevarría E., Rohaly G., Blecher C. et al. (2015) The new InsP3Kinase inhibitor BIP-4 is competitive to InsP3 and blocks proliferation and adhesion of lung cancer cells. Biochem. Pharmacol. 96, 143–150 10.1016/j.bcp.2015.05.004 25986882

[28] Ashour D.J., Pelka B., Jaaks P., Wundenberg T., Blechner C., Zobiak B. et al. (2015) The catalytic domain of inositol-1,4,5-trisphosphate 3-kinase-a contributes to ITPKA-induced modulation of F-actin. Cytoskelet Hoboken 72, 93–100 10.1002/cm.21208 25620569

[29] Tunyasuvunakool K., Adler J., Wu Z., Green T., Zielinski M., Žídek A. et al. (2021) Highly accurate protein structure prediction for the human proteome. Nature 596, 590–596 10.1038/s41586-021-03828-1 34293799 PMC8387240

[30] Mirdita M., Schütze K., Moriwaki Y., Heo L., Ovchinnikov S. and Steinegger M. (2022) ColabFold: making protein folding accessible to all. Nat. Methods 19, 679–682 10.1038/s41592-022-01488-1 35637307 PMC9184281

[31] Li L., Li C., Sarkar S., Zhang J., Witham S., Zhang Z. et al. (2012) DelPhi: a comprehensive suite for DelPhi software and associated resources. BMC Biophys. 5, 9 10.1186/2046-1682-5-9 22583952 PMC3463482

[32] Pettersen E.F., Goddard T.D., Huang C.C., Couch G.S., Greenblatt D.M., Meng E.C. et al. (2004) UCSF Chimera–a visualization system for exploratory research and analysis. J. Comput. Chem. 25, 1605–1612 10.1002/jcc.20084 15264254

